# ‘Spending the day with your Family Health Team’: rapid ethnography of a patient-centred quality improvement event

**DOI:** 10.3399/bjgpopen20X101002

**Published:** 2020-01-22

**Authors:** Katie N Dainty, Tara Kiran

**Affiliations:** 1 Research Chair, Patient-Centred Outcomes, North York General Hospital, Institute for Health Care Innovation, Toronto, Canada; 2 Assistant Professor, Institute of Health Policy Management and Evaluation, University of Toronto, Toronto, Canada; 3 Associate Professor, Department of Family and Community Medicine, St Michael's Hospital, University of Toronto, Toronto, Canada; 4 Associate Scientist, MAP Centre for Urban Health Solutions, Li Ka Shing Knowledge Institute, St Michael’s Hospital, Toronto, Canada; 5 Embedded Clinician Researcher, Health Quality Ontario, Toronto, Canada; 6 Associate Professor, Institute of Health Policy Management and Evaluation, University of Toronto, Toronto, Canada

**Keywords:** Primary health care, quality improvement, patient engagement, patient participation, qualitative research, general practice

## Abstract

**Background:**

Primary care practices have started to explore different methods of engaging with patients to advance quality improvement. This approach leverages the strengths of citizen engagement; however, there has been a lack of empirical research to understand the impact of such an approach from the patient perspective.

**Aim:**

To understand how citizen engagement can inform quality improvement in family practice.

**Design & setting:**

A single-centre, rapid ethnographic evaluation of a patient engagement event.

**Method:**

Ten thousand email invitations were sent and posters put up in Family Health Team (FHT) waiting rooms, resulting in 350 patient responses and the purposive recruitment of 36 participants. Observation and key informant interviews were used to collect data. The data corpus was analysed according to ethnographically-informed thematic analysis techniques.

**Results:**

Analysis of the full set of field notes, patient interviews, and informal conversations with the FHT staff revealed three factors that impacted on the success of the patient engagement event: setting the stage, the power of storytelling, and the value of reframing the patient role.

**Conclusion:**

The present study highlights three components of patient and public engagement approaches — the importance of setting the proper stage, storytelling as a tool, and reframing the patient role in healthcare delivery — which may provide useful guidance to those considering similar patient and public engagement events.

## How this fits in

Primary care practices have started to explore different methods of engaging with patients to advance quality improvement. To the authors’ knowledge, there has been little empirical research to understand the impact of such an approach beyond participant satisfaction. Present study findings provide empirical evidence of important considerations for those looking to organise similar patient and public engagement events.

## Introduction

Across the globe, efforts to involve patients and citizens in decision-making are well established across several domains in healthcare provision and public health^1,2^ (for example, chronic disease, acute care, and surgery). The practical aims of patient and citizen engagement are multiple, often overlapping, and sometimes conflicting; namely, to encourage change of health-related behaviours; empower citizens to take greater responsibility for their own health; control health costs; improve the quality of healthcare provision; and improve patient experience.^3^

Patient engagement to influence organisational design and governance necessitates a process for dialogue with patients about what is working well and what can be better. As per Williamson’s article on patient and citizen participation in health, the authors use the terms ‘involvement’, ‘engagement’, and ‘participation’ interchangeably in the present article *’* *to signify efforts to involve people (and patients) more actively in policy and practice* *’*.^3^ This can occur along a continuum from consultation via patient experience surveys, to involvement as patient advisors, to partnership via shared leadership of committees.^4^ Though diverse, these initiatives share the conviction that sustainable improvements in health cannot be achieved by imposing change on people but require their active participation and the consideration of their voice as equal. Much of the discussion on patient-centred care and patient engagement has focused on engagement at a clinical level, shared decision-making about treatment,^5^ and the activities of formal patient advisory councils; however, patients need to be involved more broadly, and there needs to be a better understanding of what factors make for successful engagement experiences and therefore lead to broader improvement in health care and care systems. Improved understanding of what effective patient engagement looks like; what strategies and investments can be appropriate in different settings; and how leaders throughout an organisation can advance these efforts must be discussed more openly.

Primary care practices have started to explore different methods of engaging with patients to advance quality improvement.^6^ The authors’ primary care Family Health Team (FHT) organisation chose to leverage methods of citizen engagement to develop and host a 1-day patient engagement event designed to gather feedback on core services.^7^ To the authors’ knowledge, there has been no empirical research to understand the impact of such an approach; therefore, the authors undertook a rapid ethnographic evaluation of the event to understand its impact and what context and mechanisms might relate to potential success. The authors sought to understand what citizen engagement needs to look like in order to make it a useful approach for quality improvement in family practice.

## Method

### Setting

This study evaluates a patient engagement day conducted at the St Michael’s Hospital Academic Family Health Team in Toronto, Canada. The FHT is a large, interprofessional primary care organisation with six clinic sites dispersed throughout the urban core of Toronto. The FHT serves approximately 40 000 patients from diverse backgrounds, including young urban professionals as well as immigrant families living in poverty. The vast majority of patients have health coverage through the provincial health insurance plan, which fully covers primary care visits and all medically necessary tests.

The FHT regularly surveys patients on their experience of care and uses responses to inform improvement priorities; however, the FHT wanted to engage in a richer dialogue with patients to better understand how services could be improved, and thus partnered with an external agency, MASS LBP, to organise a 1-day patient engagement event. Using citizen engagement methodology described elsewhere,^7^ the FHT sent 10 000 email invitations and put up posters in their waiting rooms inviting patients to volunteer; 350 patients responded. The FHT selected 36 of the 350 patients based on self-reported age, sex, housing status, and health status to ensure diverse perspectives from a group of patients considered to be representative of the practice population. The FHT covered transportation but not childcare costs for patient volunteers, and worked extensively to ensure each participant could attend without unreasonable burden or expense.

### Study design and population

This was a single-centre, rapid ethnographic evaluation using both observation and key informant interviews to collect data. Rapid ethnography is a collection of field methods used to provide evaluators with a reasonable understanding of participants and their activities given a limited amount of time spent in the field gathering data.^8^ It is often used in targeted community engagement events.

A convenience sample was analysed, drawn from those who participated in the patient engagement event (observation) and the subsection of the participants that provided their contact information (follow-up interview). As those attending the patient engagement event were randomly selected for participation, maximum variation sampling for the evaluation was enabled. This approach provides the opportunity to understand how an issue is experienced differently by participants in different circumstances.^9^ Verbal informed consent was obtained from all interview participants and audio-recorded at the time of the telephone interviews.

#### Recruitment and consent to participate

Patients who participated in the patient engagement event were made aware of the evaluation, and the researcher was introduced at the beginning of the session. Any participant who was not comfortable with the evaluation was asked to speak to the lead physician, and the researcher would be notified; at no point during the day did any of the participants ask to be removed from the data collection. At the end of the session, participants who were interested in participating in the follow-up interviews were asked to provide their name and primary contact phone number.

#### Data collection

Data collection for this evaluation consisted of both observation and key informant interviews. Observation was carried out during the actual event, and interviews were conducted by telephone after the event. All qualitative data was collected by a PhD-trained qualitative researcher who was not a member of the FHT or involved in the organisation of the event. The researcher did not have a prior relationship with any of the patients or staff who participated in the event.

Observations were conducted throughout the event. The researcher strategically moved around the room throughout the event in order to capture the various interactions and communications at different times during the day. During direct observations, researcher interaction with participants was minimal and non-interruptive (this was not a participant observation study). Data was collected with bullet-point jottings which were written up into detailed field notes by the researcher immediately after the event.

Post-event patient interviews lasted an average of 15 minutes (10–37 minutes) and were conducted by telephone by the researcher. The interviews followed a semi-structured format using an interview guide designed by the research team and informed by the evaluation objectives. The semi-structured format allowed the interviewee to guide the conversation while, at the same time, providing some direction around certain topics of interest. All of the interviews were assigned an ID number which was not related to the interviewee’s identity, and audio files were transcribed verbatim and de-identified (any personal identifiers removed) by an external professional transcription service.

#### Data analysis

The data collected were analysed according to ethnographically-informed thematic analysis techniques.^10^ Data analysis of the interview transcripts was carried out in parallel with data collection in order to continuously monitor emerging themes and identify areas for further exploration. All analysis was initially carried out independently by the researcher and discussed with the project team at several time points in order to ensure the analysis was grounded in the data and objective of the research. Each author provided methodological and content expertise to ensure trustworthiness of the study.^11^

The first principle of inductive analysis is to use an emergent strategy to allow the analysis to follow the nature of the data itself, with a focus on understanding the meaning of the description. The first step of the analysis was open coding. Descriptive codes were attached to segments of the text in each transcript by the researcher.^12^ The descriptive codes were then grouped in to broad topic-oriented categories and all text segments belonging to the same category were compared.^12^ The topic-oriented categories were further refined and formulated into fewer analytic categories through an inductive, iterative process. The analysis moved forward and backward as data were re-examined, with codes, categories, and patterns beginning to emerge. Observational field notes were also coded where appropriate and analysed to provide a detailed description of the context of the event. The research team had multiple discussions throughout the coding process and approved all versions of the code book prior to final coding of the dataset. Any disagreements in interpretation were settled through discussion and consensus. All coding was carried out manually without the use of computer software in order to make it easier to share thought processes with the clinical project team. Significant field notes were kept during the process of analysis in order to provide an audit trail of each step of the process and to monitor the reflexivity of the researcher.

## Results

Of the 36 attendees at the event, 12 volunteered to be interviewed. All participants who provided their contact information were interviewed. Analysis of the full set of observational field notes, patient interviews, and informal conversations with the FHT staff revealed several common messages that are relevant to understanding the experience of engaging patients in quality improvement work in primary health care. The findings discussed here are those relating to the context and experience of the event and purposefully do not include content relating to improvement opportunities and suggestions (captured elsewhere).^7^

### Setting the stage and addressing the power differential

The event was held on a sunny day in a very bright and light-filled space at the conference centre. A few senior males arrived early, all other participants arrived on time and made their way to an open space at one of the 12 available tables (tables were not assigned). The staff attending seemed unsure of what was going to happen at first; the patients seemed quite comfortable and the facilitators began engaging with participants right away, so conversations flowed from the beginning. The space was filled with natural light, there was music playing, and the initial mood was very positive.

The above observations highlight the importance of setting the stage for the event. First, the facilitator started the introduction with very purposeful language: *‘* *Today you will be spending the day with* your *F* *amily* *H* *ealth* *T* *eam* ’ (emphasis in the original). This immediately created a sense of membership, ownership, and vested interest for everyone in the room, particularly for the patients. This was important because it set the event up as more than a focus group but rather an opportunity for two-way knowledge exchange.

Second, the group participated in a geographic ’mapping where we come from‘ exercise as an ice-breaker and a unique way to start personal introductions. During the exercise, participants (both patients and staff) were asked to not only say what part of the city they were from but also what prompted them to volunteer for the event. Overwhelmingly, the answers were all very similar and related to ’getting the opportunity to give back‘; to ’form a relationship with my healthcare team‘; ’to learn and see it from the other side’; and considering St Michael’s Hospital a part of their community *’* *so it’s important to do everything we can to make it as good as possible* *‘*. These were very positive sentiments and indicated that the participants had come to *’* *invest in their investment* *’* (their words) and not just for the purposes of complaining or voicing concerns.

The final way in which a certain type of stage was set for the day was the presentation by the lead physician. Seen as a physician and figurehead at the FHT, they proceeded to tell a personal story about their experience with their small child in the emergency room just the night before, including challenges with the processes and timeliness of the care provided. During the presentation there was a palpable sense of equality between patient and staff, at least for that day, and it seemed to set the stage for the patient participants to feel safe in sharing their feedback. One participant commented:

*’* *…* *today made the doctors and nurses seem more human to us* *…* *they don’t have magic power but I feel they are hearing our voices.* *’* (Participant [P]A – female [F])

### The power of storytelling

At many of the tables, patients regularly used informal storytelling to emphasise their description of issues with the system or their suggestions for solutions. Storytelling occurred spontaneously during the group work activities. Participants were not told to share stories, rather the small group interaction seemed to encourage a sharing environment. The stories shared at the event ranged from ones of frustration, to ones of confusion, to ones that conveyed heartfelt gratitude to the team for excellent care. It was also notable that many stories ended with a phrase such as *‘I don’t understand why it has to be that way?*’. Stories do not convince by their objective truth but by their emotional impact on the listener, and there were several occasions where exemplar stories shared by the patient participants made the staff members take pause and think about certain situations in a different way.

The sharing of stories also appeared to act as a point of connection, particularly for the patient participants. While they were complete strangers to each other and had different healthcare experiences and backgrounds, they seemed to relate to each other’s stories and could use that as a common place from which to think about solutions as a group:

*’* *I haven’t had the same experiences she has had, but hearing where she is coming from helped me to think about the problem differently.* *’* (PB – male [M])

### Reframing the patient role

As patient participants began to view the lead physician as a ‘real person’ and a healthcare user themselves, there was also a lot of discussion on the need for patients to be viewed as partners with important knowledge. This includes knowledge about themselves and their bodies, as well as about their experience as patients. This drew attention to how the team engages with their patients on a regular basis; even normalised engagements like using the word ‘patient’ to refer to an individual or how they are treated at reception, to the ‘why’ and ‘how’ parts of the care process, are seen as *’*a black box‘ for patients. This puts patients in an ’only when you need to know’ position and most often in a defensive position when an experience goes awry, encouraging the hierarchical and insider-versus-outsider culture:

*’* *Even if there isn’t anything that can be done about something, it would be nice to know the reasons why things are the way they are and that they appreciate our point of view.* *’* (PC – F)

In many ways, the event allowed for a reframing of the patients as ‘insiders’ with a different perspective and in return gave them information about the ‘inside’, which allowed for a greater understanding and more thoughtful conversation around solutions. Previously, patients had only been involved in quality improvement efforts at the FHT by providing responses in a patient experience survey or by providing input into specific projects; however, in hearing how the patients spoke about their FHT, they appeared to have always considered it as ’theirs’:

*‘* [*…*] *the* *F* *amily* *H* *ealth* *T* *eam* *is a part of our community and, so it is in our best interest to make it better.* *’* (PD – M)

## Discussion

### Summary

The authors’ evaluation of a patient engagement event in a primary care practice revealed three key themes or lessons for organising an event that positively impacts participants and the organisation, including the importance of setting the proper stage and addressing the perception of the power dynamic for such an event; the use of storytelling as a tool of connection; and how such an event can reframe the role of the patient in relation to the healthcare team.

### Strengths and limitations

As with all evaluations, there are strengths and limitations inherent in this study. This was a rapid ethnography of a 1-day event with a single data collector and a convenience sample of participants, which can be seen as both a strength and a limitation. The focused event gave an opportunity for intensive data collection, however the focus was just one group of people on 1 day for a single FHT. Given the lack of empirical research in this area, the authors feel that taking a formal research approach to understanding what went on within this event, rather than only measuring participant satisfaction, is a major strength. There were certainly opportunities for selection bias and a Hawthorne-style^13^ effect to have influenced findings; however, based on the use of rigorous qualitative data collection and analysis methods, and the interactive nature of the day, the authors feel that the risk of these types of bias is low. Given the lack of empirical research in this area, this analysis contributes to the knowledge in the area of patient engagement in primary care and has revealed insights that are likely broadly applicable to other settings.

### Comparison with existing literature

Effectively engaging patients and the public in conversations about healthcare design and delivery means changing the nature of the relationships between patients and the healthcare system. Since Hippocrates, it has been recognised that there is a strong power differential between physicians and patients, which can directly impact conversations about improving quality of care. While the power differential may not always be explicitly enacted within primary care, the hierarchy is an age-old cultural residue in health care, which implicitly frames the usual relationship between patients and providers.^14^ Often this is ignored or taken for granted but addressing it head on and leveling the field, as was the case with this patient engagement event, is probably one of the most important mediators of a positive patient engagement experience.

Related to this, stories bridge the gap between the formal codified space of an organisation (roles, job descriptions, and lines of accountability) and informal uncodified space (relationships, feelings, ’unwritten rules’, and experiences).^15^ Stories offer insights into what might have been; the imaginative reconstruction of the end of a story allows us to consider different options for change.^16^ People working in healthcare organisations can be apprehensive about allowing patients to tell their stories, assuming that they will only share negative experiences. This was far from the case in the present study. The unsolicited use of storytelling here exemplifies the power of leveraging group dynamics for a deeper engagement experience. This type of engagement enables a healthcare organisation to identify areas for improvement and turn the ’this is just how we do things‘ response into a ’why do we do it that way, and can we change it?’ perspective.

This type of event seems to have created an opportunity for the relationships between patients and the healthcare team to be reframed from transactional to relational. In a study using telephone interviews with 234 adults who had seen a primary care physician within the previous 6 months, Burgoon *et al* confirmed that perceived relational communication was strongly related to affective, cognitive, and behavioral satisfaction.^13^ This shift relates not only to how the relationship is perceived during an event such as this but also in how a healthcare team transforms its interactions with patients going forward in day-to-day practice.

### Implications for research and practice

Repeated calls have been made around the world to engage and involve patients and the public more explicitly in the design and delivery of health care. Uncertainty persists around why and how to do this well; how to measure its impact; and how to involve and support a diversity of individuals, and create opportunities that enable patients to work in partnership to genuinely influence improvement decision-making. It seems that the unique environment created via this event and the unanticipated outcomes of the group dynamics have created a successful example, which other primary care organisations can look to when considering how to engage patients in quality improvement. The research approach may also encourage others to evaluate such events more formally and help the field move beyond measures of satisfaction in order to really understand the impact and outcomes of patient engagement in primary care.

This study has practical implications for practices. Practices involved in patient engagement should pay particular attention to how they can both implicitly and explicitly reduce the traditional power differential between patients and healthcare providers. Approaches range from physical setup of the room, to use of ice-breakers, to how the leaders describe themselves, the role of patients, and the purpose of the engagement. Storytelling is an effective way for patients to connect with each other and with healthcare professionals. Providing education about healthcare operations and the broader system for patients is crucial for shifting their role to one of a healthcare ‘insider’. Future research should ideally include longitudinal studies which look at the direct outcomes related to increased patient engagement in such settings and how engagement can evolve over time to improve outcomes.

Patient and family engagement approaches to health service delivery and design offer a promising pathway toward better quality health care, more efficient care, and improved population health.^3^ The findings of the present study highlight three key themes that may provide useful guidance to those considering similar patient and public engagement events: the importance of setting the proper stage; storytelling as a tool; and reframing the patient role in health care.

## References

[1] Kahssay HM, Oakley P (1999). Community involvement in health development: a review of the concept and practice.

[2] World Health Organization (1978). Declaration of Alma-Ata: international conference on primary health care, Alma-Ata, USSR, 6–12, September 1978. http://www.who.int/publications/almaata_declaration_en.pdf.

[3] Williamson L (2014). Patient and citizen participation in health: the need for improved ethical support. AmJBioeth.

[4] Gillam S, Newbould J (2016). Patient participation groups in general practice: what are they for, where are they going?. BMJ.

[5] Baker RG, Judd M, Fancott C, Maika C (2016). Creating 'engagement-capable environments' in healthcare. Patient engagement — catalyzing improvement and innovation in healthcare.

[6] LaNoue M, Mills G, Cunningham A, Sharbaugh A (2016). Concept mapping as a method to engage patients in clinical quality improvement. AnnFamMed.

[7] Kiran T, Davie S, MacLeod P (2018). Citizen engagement in primary care. Ann Fam Med.

[8] Millen DR, Boyarski A, Kellogg W. A (2000). Rapid ethnography: time deepening strategies for HCI field research. Proceedings of the 3rd conference on designing interactive systems: processes, practices, methods, and techniques.

[9] Miles MB, Huberman AM, Saldana J (1994). Qualitative data analysis.

[10] Braun V, Clarke V, Cooper H, Camic P. M, Long D. L (2012). Thematic analysis. APA handbook of research methods in psychology, Vol 2: Research designs: Quantitative, qualitative, neuropsychological, and biological.

[11] Krefting L (1991). Rigor in qualitative research: the assessment of trustworthiness. AmJOccupTher.

[12] Kuckartz U, Kelle U (1995). Case-oriented quantification. Computer-aided qualitative data analysis: theory, methods, and practice.

[13] Oswald D, Sherratt F, Smith S (2014). Handling the Hawthorne effect: the challenges surrounding a participant observer. Review of Social Studies.

[14] Muller J, Crabtree BF, Miller WL (1999). Narrative approaches to qualitative research in primary care. Doing qualitative research.

[15] Burgoon JK, Pfau M, Parrott R (1987). Relational communication, satisfaction, compliance‐gaining strategies, and compliance in communication between physicians and patients. Commun Monogr.

[16] Ocloo J, Matthews R (2016). From tokenism to empowerment: progressing patient and public involvement in healthcare improvement. BMJ Qual Saf.

